# Supplementary material for: Moisture-induced autonomous surface potential oscillations for energy harvesting
Source: Nat Commun. 2021 Sep 6;12:5287. doi: 10.1038/s41467-021-25554-y (PMC8421362; doi:10.1038/s41467-021-25554-y)
Supplement: Supplementary file 1 — Editorial Summary [file 41467_2021_25554_MOESM1_ESM.docx]

Moisture-induced energy generation is a potential green energy power source. Here, the authors report a moisture-induced autonomous surface potential oscillation phenomenon and apply it to the demonstration of energy harvesters with long persistence time and good energy density
