# Supplementary material for: Moisture-induced autonomous surface potential oscillations for energy harvesting
Source: Nat Commun. 2021 Sep 6;12:5287. doi: 10.1038/s41467-021-25554-y (PMC8421362; doi:10.1038/s41467-021-25554-y)
Supplement: Supplementary file 2 — Supplementary Information [file 41467_2021_25554_MOESM2_ESM.pdf]

Supplementary Materials For

Moisture-induced autonomous surface potential oscillations for energy harvesting

Yu Long[1], Peisheng He[1], Zhichun Shao[1], Zhaoyang Li[2], Han Kim[3], Archie Mingze Yao[4], Yande Peng[1], Renxiao Xu[1], Christine Heera Ahn[1], Seung-Wuk Lee[3], Junwen Zhong[2*] and Liwei Lin[1*]

1 Department of Mechanical Engineering, University of California Berkeley, Berkeley, California, 94720, USA.

2 Department of Electromechanical Engineering and Centre for Artificial Intelligence and Robotics, University of Macau, Macau SAR, 999078, China

3 Department of Bioengineering, University of California Berkeley, Berkeley, California, 94720, USA

4 Department of Engineering Mechanics, Tsinghua University, Beijing, 100084, China

junwenzhong@um.edu.mo

lwlin@berkeley.edu

Supplementary Figures

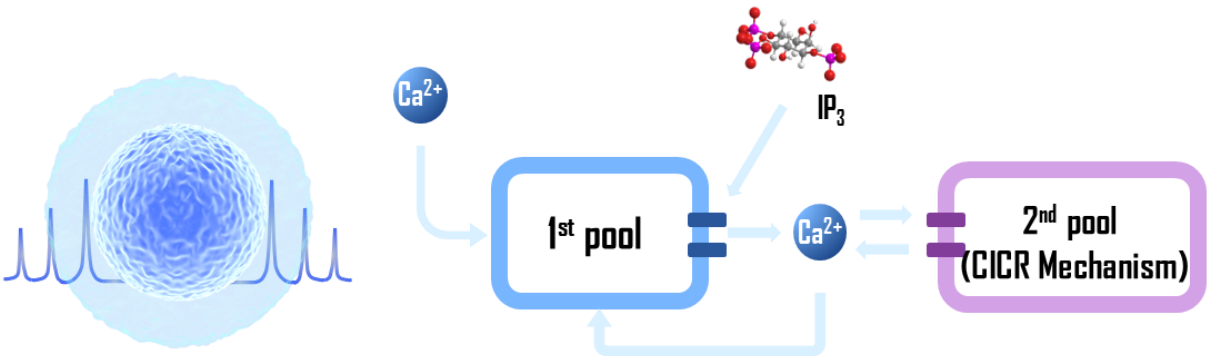

Supplementary Figure 1. A schematic diagram illustrating the Calcium-induced Ca^{2+} release (CICR) process to induce the Ca^{2+} concentration oscillation in a living cell.

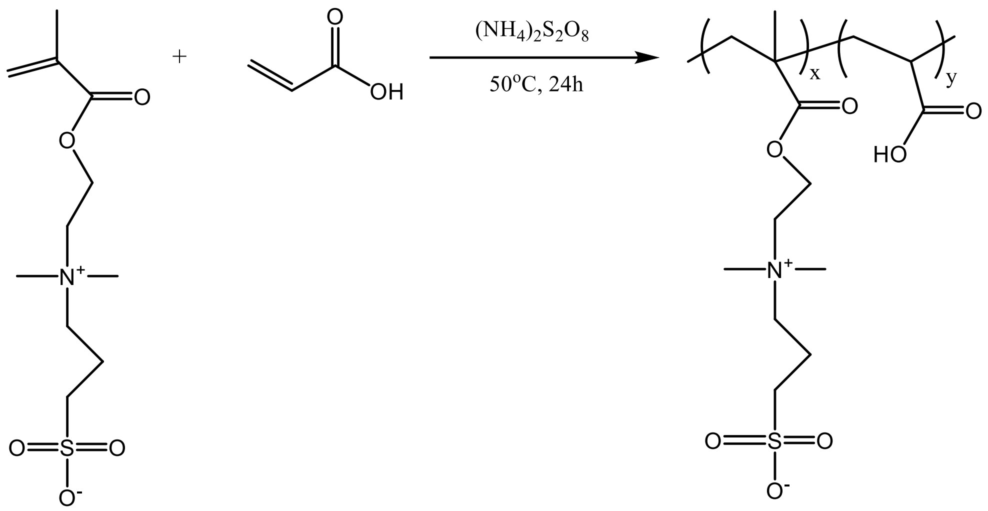

Supplementary Figure 2. The synthesis process of P(MEDSAH-co-AA) polymer.

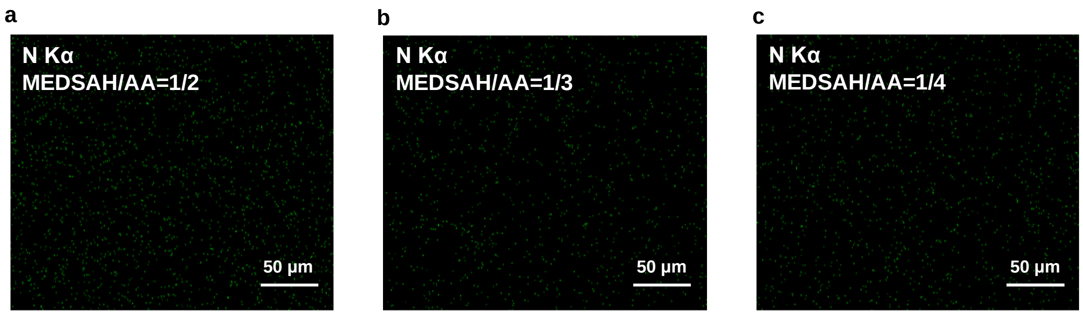

Supplementary Figure 3. N-Kα EDX results of P(MEDSAH-co-AA) polymers with different compositions: **a** MEDSAH/AA=1/2; **b** MEDSAH/AA=1/3; and **c** MEDSAH/AA=1/4.

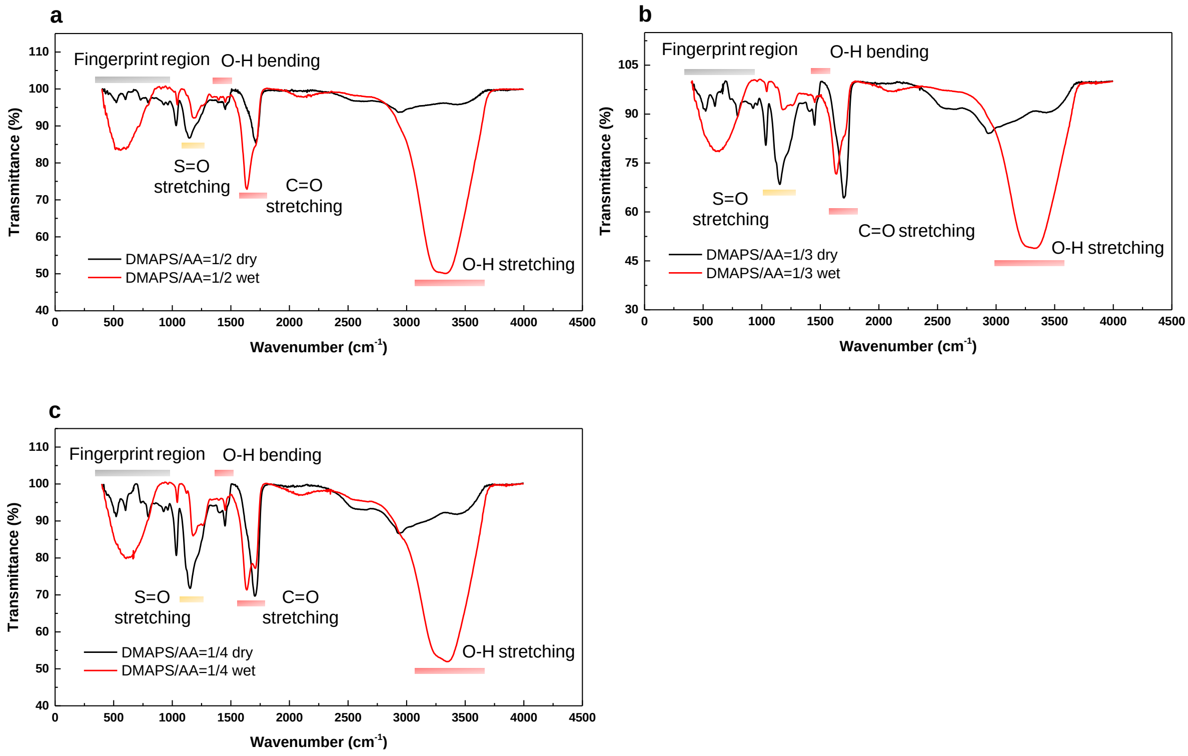

Supplementary Figure 4. ATR-FT-IR results of dry and wet P(MEDSAH-co-AA polymers with different compositions: **a** MEDSAH/AA=1/2; **b** MEDSAH/AA=1/3; **c** MEDSAH/AA=1/4.

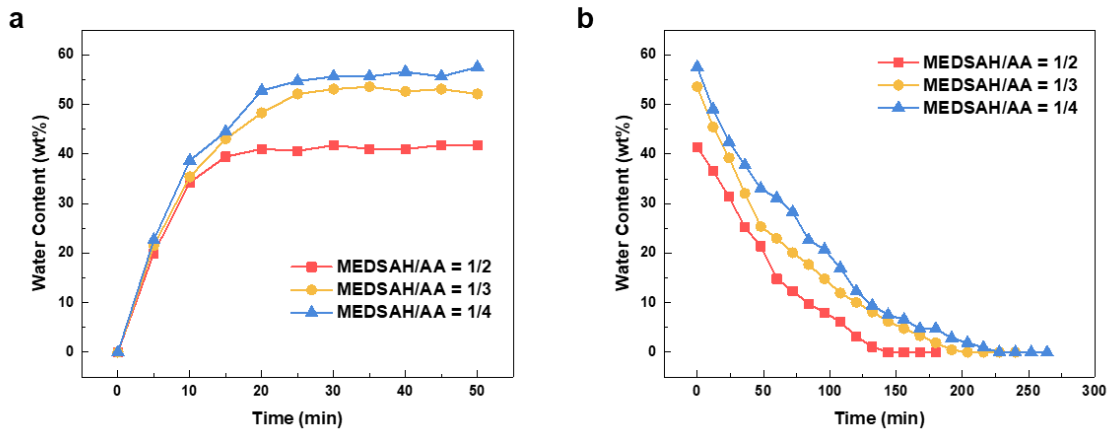

Supplementary Figure 5. The water content of polymers with different compositions during: **a** the hydration and **b** the dehydration process. P(MEDSAH-co-AA polymer containing more -COOH can absorb more moisture at a faster speed in **a** and maintain higher moisture concentrations in **b**.

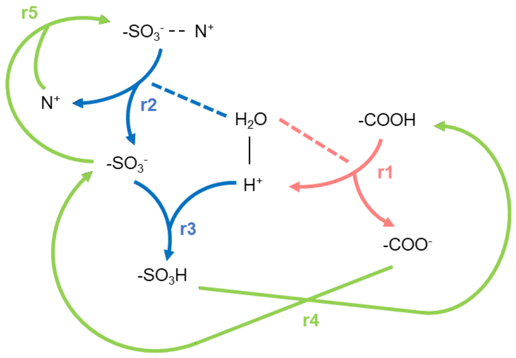

Supplementary Figure 6. Summary of the proposed “proton oscillation” mechanism by different chemical reactions: r1 (positive feedback in the red color), r2 and r3 (negative feedback in the blue color), r4 and r5 (returning to initial state in the green color). The dash lines suggest the participation of water molecules in r1 and r2.

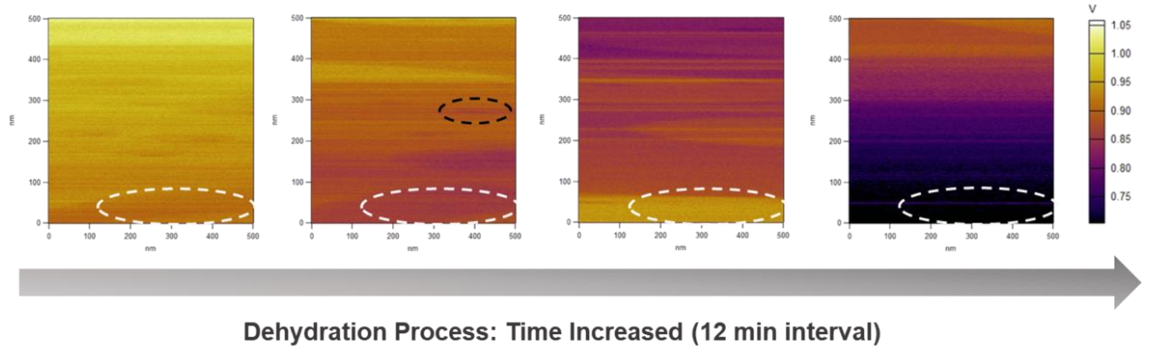

Supplementary Figure 7. Kelvin probe force microscopy (KPFM) for surface potential oscillation results at the same surface location of a prototype polymer during the moisture activation and dehydration process. Each test needs about 12 minutes to complete and the white elliptical area highlights the local surface potential oscillations. Other areas have also experience surface potential oscillations with slightly different surface potential values.

After saturating the P(MEDSAH-co-AA) polymer with water at 41 wt%, the Kelvin probe force microscopy (KPFM) is used to measure the surface potential at a fixed area of 500×500 nm^2 as depicted in Supplementary Fig. 7. In general, the average surface potential is found to gradually decrease over time. However, the time-varying oscillations in a short period of time are also observed. For example, in the white elliptical area in Supplementary Fig. 7, the surface potential is found to oscillate during a period of every 12 minutes (this test is limited by the scanning speed of the KPFM) from 0.92 V, 0.85 V, 0.96 V, to 0.74 V, sequentially. Even though limited by scanning speed, KPFM analysis shows the general oscillation tendency of the surface potential. The other areas also experience similar but different surface potential oscillations as observed.

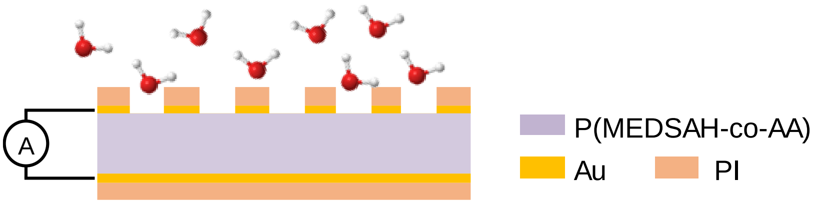

Supplementary Figure 8. The cross-sectional view of the energy harvester device.

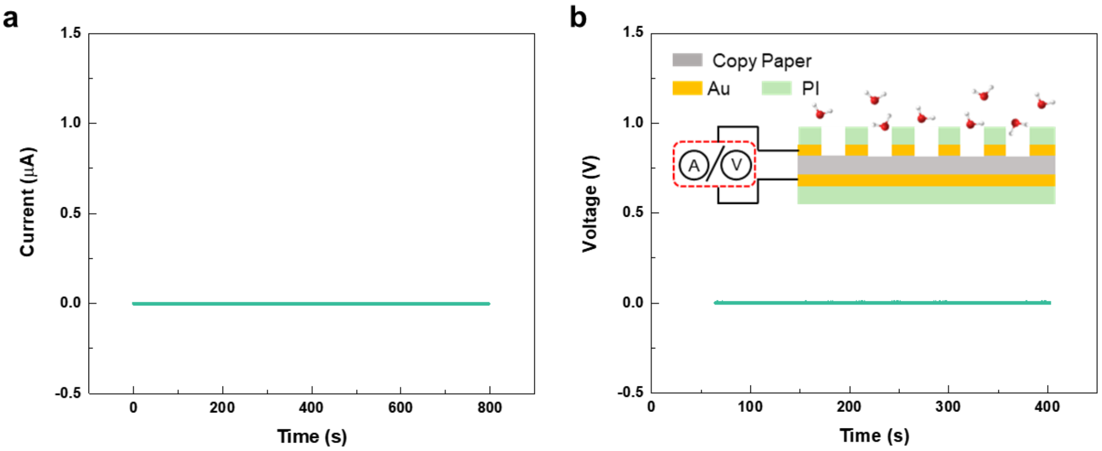

Supplementary Figure 9. **a** Close-circuit current and **b** open-circuit voltage *vs.* time results of a prototype device by using a copy paper instead of the P(MEDSAH-co-AA) polymer. No visible electrical outputs are identified.

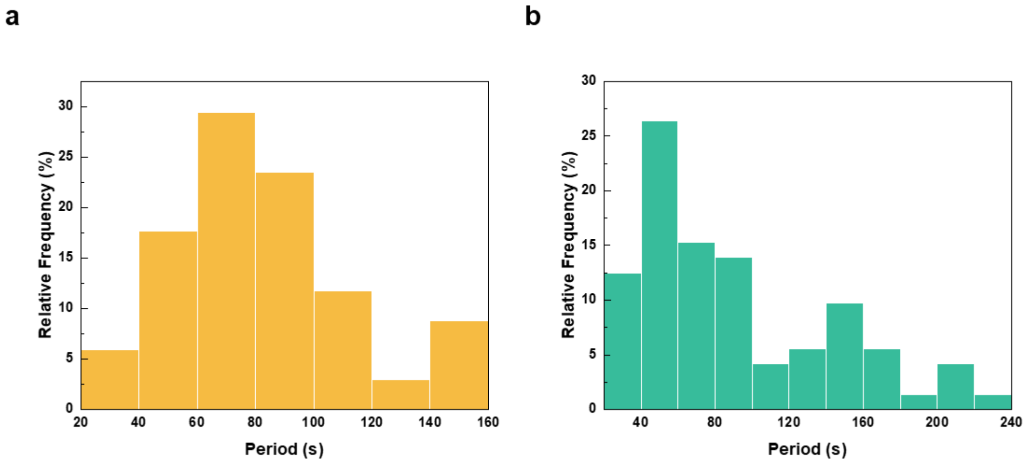

Supplementary Figure 10. The histogram of energy generation cycle period of a prototype energy harvester: **a** energy generation period; and **b** low electrical output periods (noises).

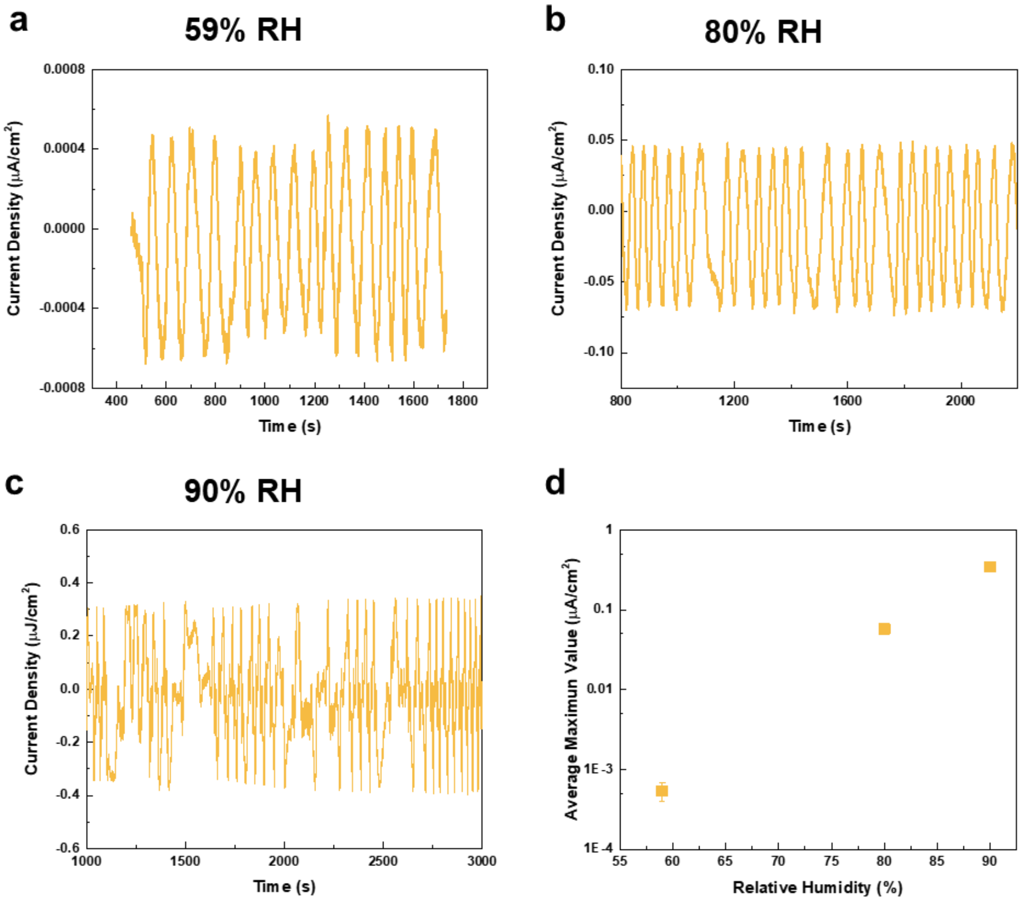

Supplementary Figure 11. The measured output current density versus time of the prototype energy harvester under various humidity levels of **a** 59% RH, **b** 80% RH, and **c** 90% RH. **d** Average maximum current density versus relative humidity.

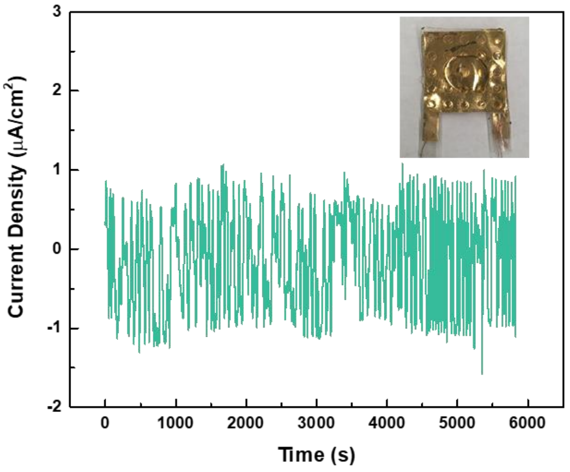

Supplementary Figure 12. The output current vs. time plot by placing a water droplet on the device.

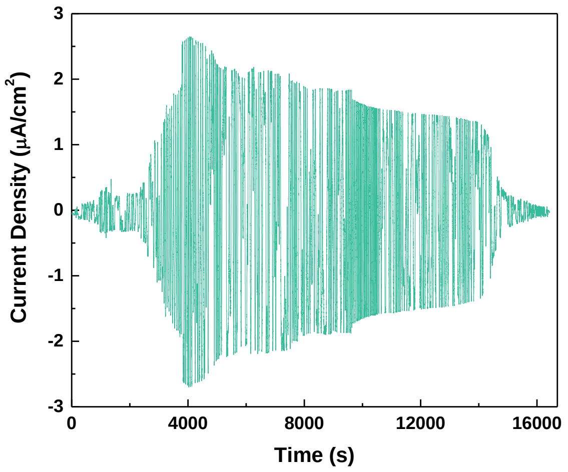

Supplementary Figure 13. The current output under continuous moisture feeding without the dehydration process.

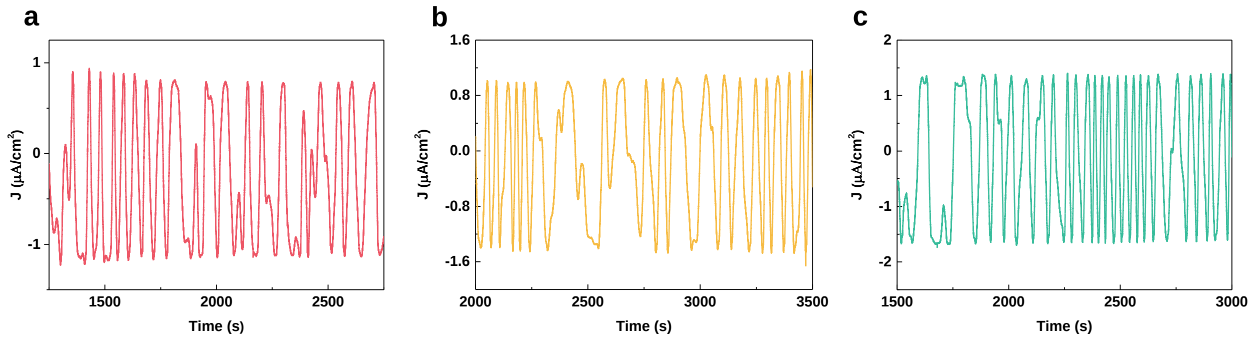

Supplementary Figure 14. Output current density versus time of energy harvesters with different polymer compositions: **a** MEDSAH/AA=1/2; **b** MEDSAH/AA=1/3; **c** MEDSAH/AA=1/4.

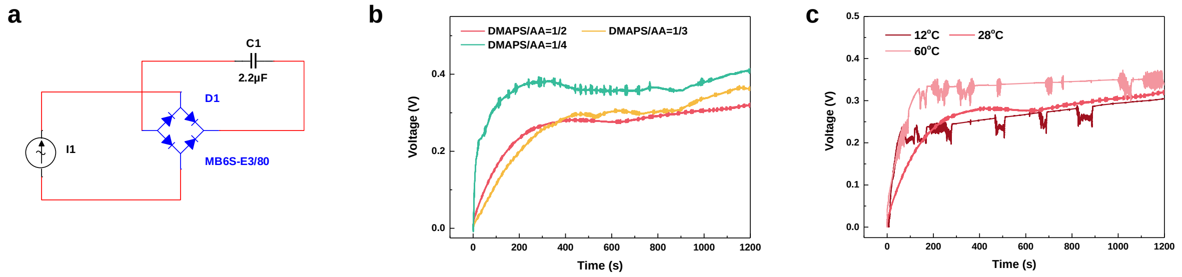

Supplementary Figure 15. **a** Electrical circuit used to charge the capacitor for measuring the DC output voltage; **b** voltage versus time plot of energy harvesters with different polymer compositions ("reference" refers to the outside noise); **c** voltage versus time plot of the energy harvester (MEDSAH/AA=1/4) under different working temperatures.

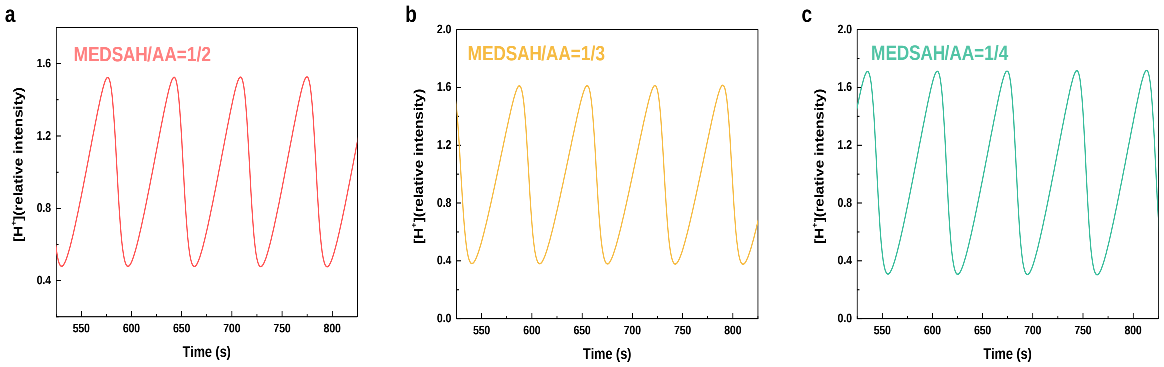

Supplementary Figure 16. Proton oscillation simulation results of polymers with different compositions: **a** MEDSAH/AA=1/2; **b** MEDSAH/AA=1/3; **c** MEDSAH/AA=1/4.

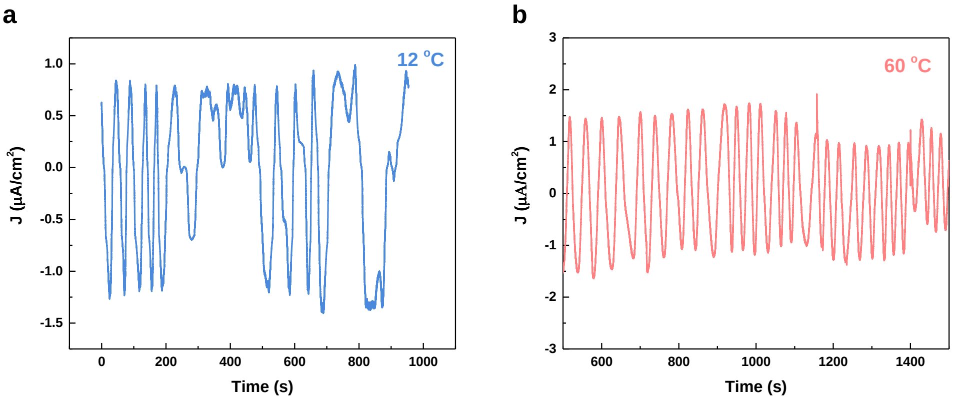

Supplementary Figure 17. Measured output current versus time of the energy harvester (MEDSAH/AA=1/2) at **a** 12 °C and **b** 60 °C.

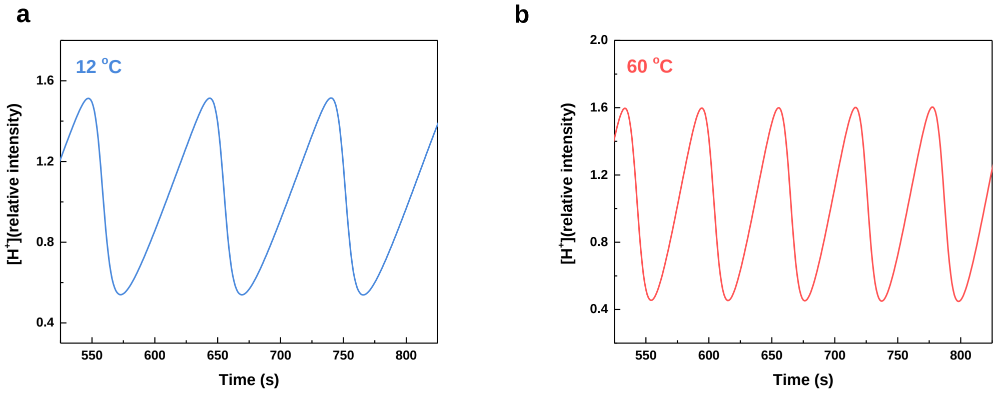

Supplementary Figure 18. Simulation results of proton oscillation versus time for polymer (MEDSAH/AA=1/2) under **a** 12 °C and **b** 60 °C.

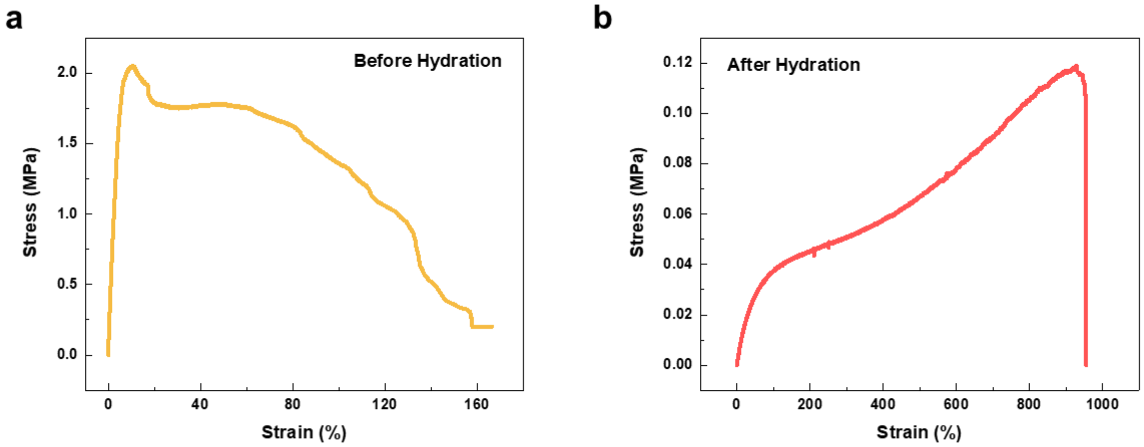

Supplementary Figure 19. The measured stress-strain curves of the polymer **a** before and **b** after the hydration process. The calculated Young's modulus values are 30.6 MPa and 66.8 kPa before and after hydration, respectively.

Supporting Explanation 1: Kinetic Simulation:

Kinetic analysis of the chemical reactions is performed based on the 5 key reactions in the system:

$r_1: -COOH == H^+ + -COO^-$

$r_2: -SO_3^-N^+ == -SO_3^- + -N^+$

$r_3: -SO_3^- + H^+ == -SO_3H$

$r_4: -SO_3H + -COO^- == -SO_3^- + -COOH$

$r_5: -SO_3^- + -N^+ == -SO_3^-N^+$

The reaction rate constants are added as:

$v_{r1} = k_1[-COOH]$

$v_{r2} = k_2[-SO_3^-N^+]$

$v_{r3} = k_3[-SO_3^-][H^+]$

$v_{r4} = k_4[-SO_3H][-COO^-]$

$v_{r5} = k_5[-SO_3^-][-N^+]$

Therefore, the concentration gradient of each species with respect to time can be expressed as:

$$\frac{d[H^+]}{dt} = k_1[-COOH] - k_3[-SO_3^-][H^+] \quad (1)$$
$$\frac{d[-COOH]}{dt} = -k_1[-COOH] + k_4[-SO_3H][-COO^-] \quad (2)$$
$$\frac{d[-SO_3^-]}{dt} = k_2[-SO_3^-N^+] - k_3[-SO_3^-][H^+] + k_4[-SO_3H][-COO^-] \quad (3)$$
$$\frac{d[-SO_3^-N^+]}{dt} = -k_2[-SO_3^-N^+] + k_5[-SO_3^-][-N^+] \quad (4)$$

Furthermore, $[-N^+]$, $[-SO_3H]$ and $[-COO^-]$ are corelated by the conservation of matters:

$[-N^+] + [-SO_3^-N^+] = [-SO_3^-N^+] + [-SO_3H] + [-SO_3^-] = c_{N+SO3-}$

$[-COOH] + [-COO^-] = c_{carboxylic\ acid}$

The concentration of water, $[H_2O]$, is controlled by v_{in} and v_{out} as:

$$v_{in} = v_{out}\frac{[H^+]}{[H^+]_0}$$

where $[H^+]$ is the concentration of hydrogen cation and $[H^+]_0$ is the initial concentration. The concentration of water versus time can be expressed as:

$$\frac{d[H_2O]}{dt} = -v_{out} + v_{in}\frac{[H^+]}{[H^+]_0} \quad (5)$$

The water concentration affects the breaking of ionization bonds such that a factor is introduced in the reaction rate constant of r2 when the concentration of water, $[H_2O]$, is lower than the threshold value $c_{threshold}$:

$$k_2 = \begin{cases} e^{-0.5(c_{threshold}-[H_2O])}\, k_2^0, & when\ c_{threshold} > [H_2O] \\ k_2^0, & when\ c_{threshold} \le [H_2O] \end{cases} \quad (6)$$

To numerically solve the differential equations (1)-(5), the explicit 1st-upwind scheme is adopted:

$$\frac{dA}{dt} \approx \frac{A(t+\Delta t) - A(t)}{\Delta t} \quad (7)$$

By matching with experimental data, an optimal set of constants: $k_1 = 0.0000962$, $k_2^0 = 0.0949$, $k_3 = 0.1118$, $k_4 = 0.0871$, $k_5 = 0.0936$, timestep $\Delta t = 0.01$, $v_{in} = 0.94$, $c_{threshold} = 200$ have been identified for the system, while c_{N+SO3-} and $c_{carboxylic\ acid}$ varies according to different proportions of the total mass which is fixed as a constant at 150×10^5. Other sets of constants will produce different results in operation periods which deviate more from experimental observations. For example, by setting $k_1 = 0.000163$, $k_2^0 = 0.1606$, $k_3 = 0.1892$, $k_4 = 0.1474$, $k_5 = 0.1584$, the oscillation period is decreased to 57s. By setting $k_1 = 0.000074$,

$k_2^0 = 0.073$, $k_3 = 0.086$, $k_4 = 0.067$, $k_5 = 0.072$, the oscillation period is increased to 97s. Supplementary Table 1 summarizes these results.

Supplementary Table 1. Summary of the kinetic constants

Period (s)	k_1	k_2^0	k_3	k_4	k_5
**57**	0.000163	0.1606	0.1892	0.1474	0.1584
**70**	0.0000962	0.0949	0.1118	0.0871	0.0936
**97**	0.000074	0.073	0.086	0.067	0.072

The initial concentration of H^+, $[H^+]_0$ has a positive correlation with $c_{carboxylic\ acid}$ and other initial concentration is set as follows:

$$[-SO_3^-]_0 = 0$$

$$[H_2O]_0 = 170$$

The calculation script is written in python3 and resulting data are normalized by the initial proton concentration. Based on experimental results, the oscillation period is adjusted to about 70 s, by adjusting kinetic constants. It is also found that the oscillation period has different sensitivity on the kinetic constants. For example, k_2^0 and k_4 have minimum influences on the oscillation period (Supplementary Figure 20), while k_1, k_3 and k_5 can affect the oscillation period strongly (Supplementary Fig. 21).

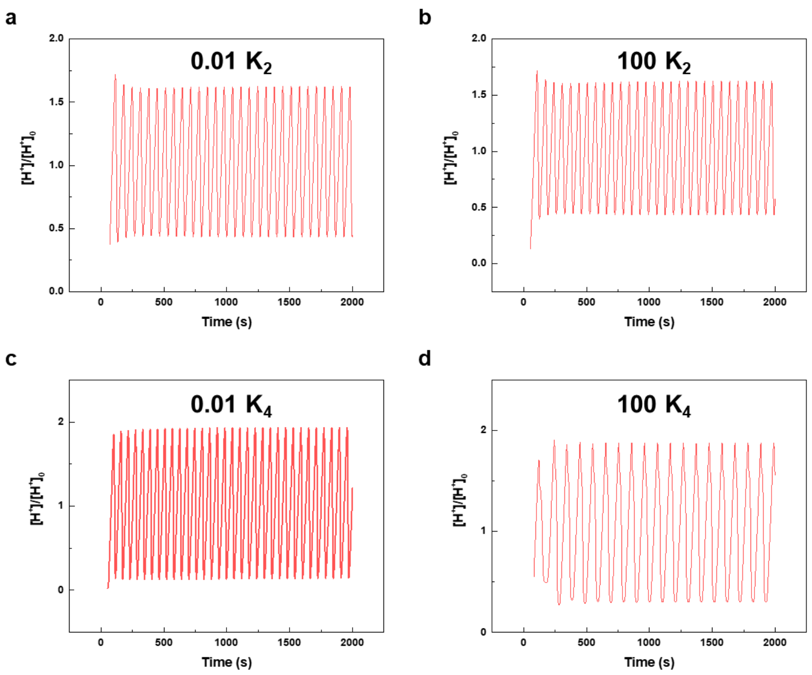

Supplementary Figure 20. Proton concentration versus time simulation results. **a** and **b** k_2^0 values are reduced 100 times and increased 100 times, respectively. In both cases, proton concentration oscillations are observed with a period around 70 s without obvious changes. **c** and **d** k_4 values are reduced 100 times (with a period about 60 s) and increased 100 times (with a period about 100 s), respectively. In both cases, proton concentration oscillations are still observed with minor changes in the time periods.

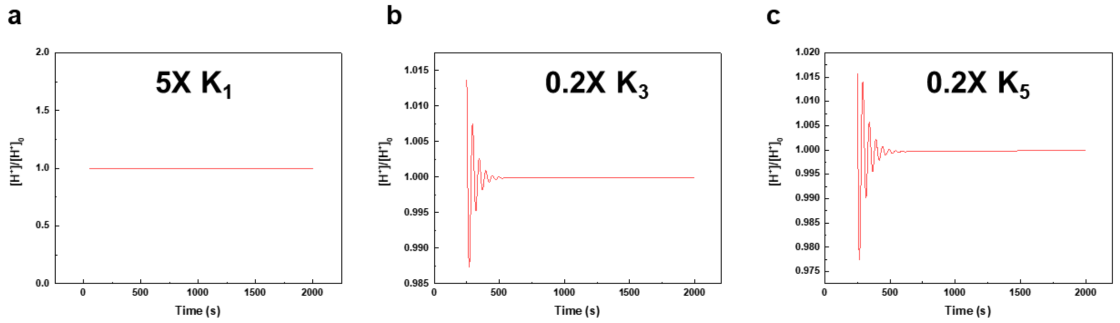

Supplementary Figure 21. Proton concentration versus time simulation results. **a** By increasing the k_1 value 5 times larger, the proton concentration oscillation disappears completely. **b** By reducing the k_3 value 5 times smaller, the proton concentration oscillation disappears very soon. **c** By reducing the k_5 value 5 times smaller, the proton concentration oscillation disappears very soon.

Supporting Explanation 2: System Oscillation Study

The stability matrix of the system is established by following standard methods[1,2] and simplifying the parameter notation as below:

$[-\mathrm{COOH}]: x_1$; $[\mathrm{H}^+]: x_2$; $[-\mathrm{COO}^-]: x_3$; $[-\mathrm{SO_3H}]: x_4$; $[-\mathrm{SO_3}^-\mathrm{N}^+]: x_5$;

$[-\mathrm{SO_3}^-]: x_6$; $[-\mathrm{N}^+ -]: x_7$;

$Total\ [-\mathrm{SO_3}^-\mathrm{N}^+]: A$;

$Total\ [-\mathrm{COOH}]: B$;

The relationships of these variables can be derived as:

$$\begin{cases} x_7 + x_5 = A \\ x_5 + x_4 + x_6 = A \\ x_1 + x_3 = B \end{cases} \tag{8}$$

$$\begin{cases} \dfrac{dx_2}{dt} = k_1x_1 - k_3x_6x_2 \\ \dfrac{dx_1}{dt} = -k_1x_1 + k_4x_4x_3 \\ \dfrac{dx_6}{dt} = k_2x_5 - k_3x_6x_2 + k_4x_4x_3 \\ \dfrac{dx_5}{dt} = -k_2x_5 + k_5x_6x_7 \end{cases} \tag{9}$$

From Eq.(8), x_7 can be derived by x_5; x_3 can be derived by x_1, and x_4 can be derived by $x_1$and x_6, and the seven variables (x_1, x_2, x_3, x_4, x_5, x_6, x_7) can be reduced to four variables (x_1, x_2, x_5, x_6) as

$$\begin{cases} \dfrac{dx_2}{dt} = k_1x_1 - k_3x_6x_2 \\ \dfrac{dx_1}{dt} = -k_1x_1 - k_4Bx_5 - k_4Bx_6 - k_4Ax_1 + k_4x_1x_5 + k_4x_1x_6 + k_4AB \\ \dfrac{dx_6}{dt} = k_2x_5 - k_3x_6x_2 - k_4Bx_5 - k_4Bx_6 - k_4Ax_1 + k_4x_1x_5 + k_4x_1x_6 + k_4AB \\ \dfrac{dx_5}{dt} = -k_2x_5 + k_5Ax_6 - k_5x_5x_6 \end{cases} \tag{10}$$

Small perturbations are added for the system oscillation study:

$$\begin{cases} x_1 = x_{10} + \delta_1 \\ x_2 = x_{20} + \delta_2 \\ x_5 = x_{50} + \delta_5 \\ x_6 = x_{60} + \delta_6 \end{cases} \tag{11}$$

where δ_i are small variations of each corresponding variables and the dynamic equations can be written in the following form (neglecting the constant terms):

$$\begin{bmatrix} \frac{dx_2}{dt} \\ \frac{dx_1}{dt} \\ \frac{dx_6}{dt} \\ \frac{dx_5}{dt} \end{bmatrix} = \begin{bmatrix} -k_3x_{60} & k_1 & -k_3x_{20} & 0 \\ 0 & -k_1 - k_4A + k_4x_{50} + k_4x_{60} & -k_4B + k_4x_{10} & -k_4B + k_4x_{10} \\ -k_3x_{60} & -k_4A + k_4x_{50} + k_4x_{60} & -k_4B + k_4x_{10} & k_2 - k_4B + k_4x_{10} \\ 0 & 0 & k_5A - k_5x_{50} & -k_2 - k_5x_{60} \end{bmatrix} \begin{bmatrix} \delta_2 \\ \delta_1 \\ \delta_6 \\ \delta_5 \end{bmatrix} \tag{12}$$

The stability matrix of this system is:

$$\begin{bmatrix} -k_3x_{60} & k_1 & -k_3x_{20} & 0 \\ 0 & -k_1 - k_4A + k_4x_{50} + k_4x_{60} & -k_4B + k_4x_{10} & -k_4B + k_4x_{10} \\ -k_3x_{60} & -k_4A + k_4x_{50} + k_4x_{60} & -k_4B + k_4x_{10} & k_2 - k_4B + k_4x_{10} \\ 0 & 0 & k_5A - k_5x_{50} & -k_2 - k_5x_{60} \end{bmatrix} \tag{13}$$

Here, k_i, A and B are parameters in the simulation, and $x_{10,20,50,60}$ are the solutions of:

$$\begin{cases} \frac{dx_2}{dt} = k_1x_1 - k_3x_6x_2 = 0 \\ \frac{dx_1}{dt} = -k_1x_1 - k_4Bx_5 - k_4Bx_6 - k_4Ax_1 + k_4x_1x_5 + k_4x_1x_6 + k_4AB = 0 \\ \frac{dx_6}{dt} = k_2x_5 - k_3x_6x_2 - k_4Bx_5 - k_4Bx_6 - k_4Ax_1 + k_4x_1x_5 + k_4x_1x_6 + k_4AB = 0 \\ \frac{dx_5}{dt} = -k_2x_5 + k_5Ax_6 - k_5x_5x_6 = 0 \end{cases} \tag{14}$$

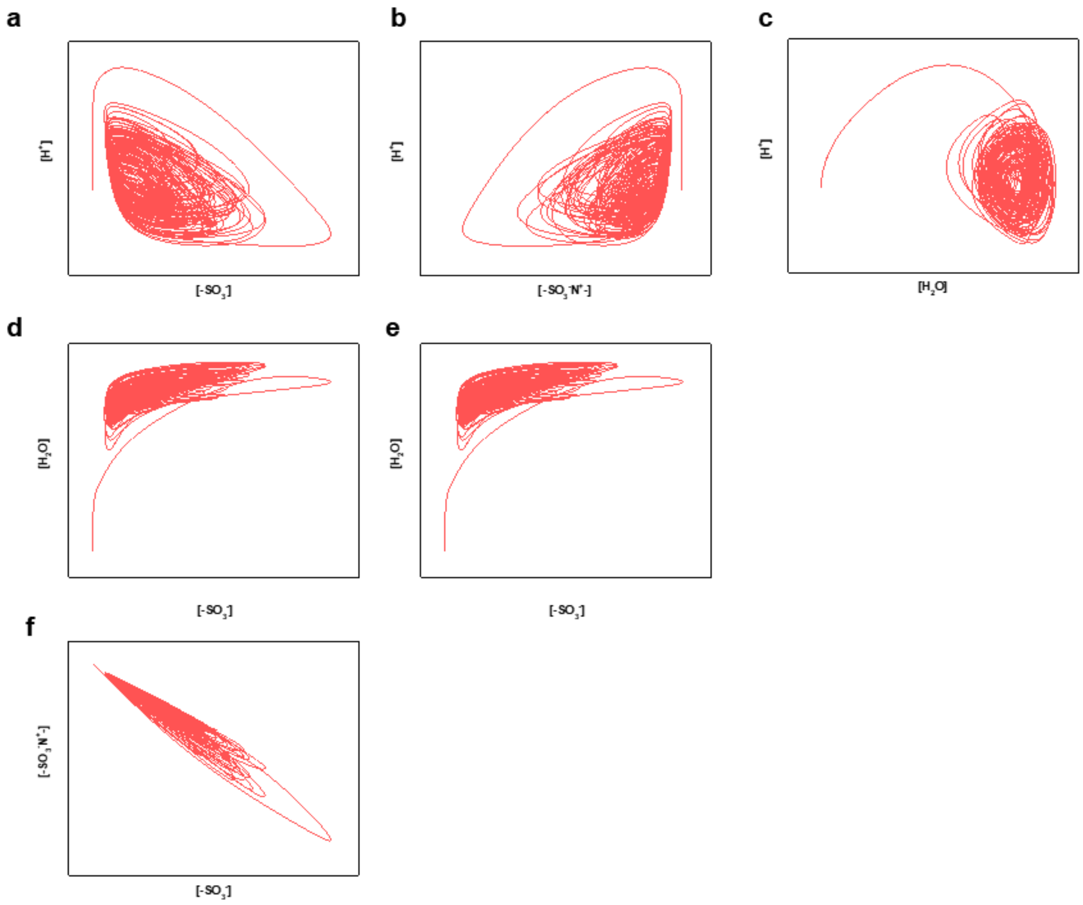

Supplementary Figure 22. The phase portraits of the system, showing the oscillating and chaotic behaviors. The above six figures are projections on different concentration coordinate systems in the four-dimensional space. The projection planes are **a** H^+ and $-SO_3^-$; **b** H^+ and $-SO_3^-N^+$; **c** H^+ and H_2O; **d** H_2O and $-SO_3^-$; **e** H_2O and $-SO_3^-N^+$; **f** $-SO_3^-N^+$ and $-SO_3^-$.

It is found that the above equation set has no solutions and this means there is no stability matrix, such that the system is chaotic with hidden attractors[3]. Supplementary Fig. 22 are the phase portraits for a simulation time of 5,000 s to show the behavior of the system and the projections of those attractors. The parameters used in this exemplified study are those listed in Supporting Explanation 1 (with an oscillation period of 70 s). Since the system is chaotic, the phase portraits will change and cannot be predicted with a different set of parameters.

Supporting Explanation 3: Analogy to Ca^{2+} oscillation:

Ca^{2+} oscillation	This work	Similarity
IP_3	H_2O	Start the oscillation
Ca^{2+}	H^+	Oscillated signal
1st pool	-COOH	In positive feedback(s)
2nd pool	$-SO_3^-$	In negative feedback(s)

IP_3 plays a role similar to H_2O to start the oscillation process; the 1st pool and -COOH groups can execute the positive feedback part of the oscillation procedure, and the 2nd pool and $-SO_3^-$ groups play the role to complete the negative feedback part of the oscillation process.

Supplementary Table 2. Measured potential (Reading) and the corresponding pH values.

pH	Reading(mV)
2.51	746.3
3.57	686.4
4.47	622.2
4.78	600.7

Supplementary Reference:

1. Pham, V. T. et al. Constructing a novel no-equilibrium chaotic system. *Int. J. Bifurc. Chaos* **24**, 1450073 (2014).
2. Pham, V. T. *et al.* Coexistence of hidden chaotic attractors in a novel no-equilibrium system. *Nonlinear Dyn.* **87**, (2017).
3. Panahi, S. et al. A new four-dimensional chaotic system With no equilibrium point. in *Recent Advances in Chaotic Systems and Synchronization* 63–76 (Elsevier, 2019).
