# Supplementary material for: Moisture-induced autonomous surface potential oscillations for energy harvesting
Source: Nat Commun. 2021 Sep 6;12:5287. doi: 10.1038/s41467-021-25554-y (PMC8421362; doi:10.1038/s41467-021-25554-y)
Supplement: Supplementary file 4 — Description of Additional Supplementary Files [file 41467_2021_25554_MOESM4_ESM.pdf]

Description of Additional Supplementary Files

File Name: Supplementary Movie 1
Description: COMSOL simulation of AC electricity generation process of the moisture-induced energy harvester.

File Name: Supplementary Movie 2
Description: The moisture-induced energy harvester is placed in a bathroom full of moisture to light up an LCD.
